# The Difference of Gut Microbiota and Their Correlations With Urinary Organic Acids Between Autistic Children With and Without Atopic Dermatitis

**DOI:** 10.3389/fcimb.2022.886196

**Published:** 2022-06-21

**Authors:** Ru-ping Hong, Yue-ying Hou, Xin-jie Xu, Ji-dong Lang, Yun-feng Jin, Xiao-feng Zeng, Xuan Zhang, Geng Tian, Xin You

**Affiliations:** ^1^ Department of Rheumatology and Clinical Immunology, Peking Union Medical College Hospital, Chinese Academy of Medical Sciences & Peking Union Medical College, Beijing, China; ^2^ Medical Science Research Center, Research Center for Translational Medicine, Department of Scientific Research, Peking Union Medical College Hospital, Beijing, China; ^3^ Geneis Beijing Co., Ltd., Beijing, China; ^4^ Beijing Herun Clinic, Beijing, China; ^5^ Key Laboratory of Rheumatology & Clinical Immunology, Ministry of Education, Beijing, China; ^6^ National Clinical Research Center for Dermatologic and Immunologic Diseases (NCRC-DID), Beijing, China; ^7^ Autism Special Fund, Peking Union Medical Foundation, Beijing, China

**Keywords:** autism, atopic dermatitis, gut microbiota, organic acids, mitochondrial dysfunction

## Abstract

Autism is a kind of biologically based neurodevelopmental condition, and the coexistence of atopic dermatitis (AD) is not uncommon. Given that the gut microbiota plays an important role in the development of both diseases, we aimed to explore the differences of gut microbiota and their correlations with urinary organic acids between autistic children with and without AD. We enrolled 61 autistic children including 36 with AD and 25 without AD. The gut microbiota was sequenced by metagenomic shotgun sequencing, and the diversity, compositions, and functional pathways were analyzed further. Urinary organic acids were assayed by gas chromatography–mass spectrometry, and univariate/multivariate analyses were applied. Spearman correlation analysis was conducted to explore their relationships. In our study, AD individuals had more prominent gastrointestinal disorders. The alpha diversity of the gut microbiota was lower in the AD group. LEfSe analysis showed a higher abundance of *Anaerostipes caccae*, *Eubacterium hallii*, and *Bifidobacterium bifidum* in AD individuals, with *Akkermansia muciniphila*, *Roseburia intestinalis*, *Haemophilus parainfluenzae*, and *Rothia mucilaginosa* in controls. Meanwhile, functional profiles showed that the pathway of lipid metabolism had a higher proportion in the AD group, and the pathway of xenobiotics biodegradation was abundant in controls. Among urinary organic acids, adipic acid, 3-hydroxyglutaric acid, tartaric acid, homovanillic acid, 2-hydroxyphenylacetic acid, aconitic acid, and 2-hydroxyhippuric acid were richer in the AD group. However, only adipic acid remained significant in the multivariate analysis (OR = 1.513, 95% CI [1.042, 2.198], P = 0.030). In the correlation analysis, *Roseburia intestinalis* had a negative correlation with aconitic acid (r = -0.14, P = 0.02), and the latter was positively correlated with adipic acid (r = 0.41, P = 0.006). Besides, the pathway of xenobiotics biodegradation seems to inversely correlate with adipic acid (r = -0.42, P = 0.18). The gut microbiota plays an important role in the development of AD in autistic children, and more well-designed studies are warranted to explore the underlying mechanism.

## Introduction

Autism spectrum disorder (ASD) is a kind of neurodevelopmental condition and troubles a lot of children in the world, with a prevalence up to 1%–2% (Liu et al., 2022). ASD is characterized by a deficit in social communication and interaction and the restrictive, repetitive pattern of behavior (Lai et al., 2014). What is worse, the coexistence of neuropsychiatric disorders and allergic diseases is not uncommon (Simpson, 2012; Miyazaki et al., 2017). In a large cross-sectional study, ASD children showed a higher odds ratio in association with skin allergy compared with children without ASD (Xu et al., 2018). Billeci et al. retrospectively analyzed 18 studies to assess the relationship between ASD and atopic dermatitis (AD). They found that ASD was positively associated with AD compared with typically developing controls, and vice versa (Billeci et al., 2015). Moreover, the frequencies of AD in ASD varied, ranging from 7% to 64.2%. Atopic dermatitis is a chronically inflammatory skin disease featured with intense itching and relapsing eczema-like skin lesions and is one of the most common skin diseases in children (Weidinger et al., 2018). It is generally accepted that epidermal barrier abnormalities and T-cell abnormal activation are involved in the development of AD, and it shows a relationship with some autoimmune diseases especially those affecting the skin, the gastrointestinal tract, and the connective tissue, such as systemic lupus erythematosus (Narla and Silverberg, 2019; Ivert et al., 2021). AD mostly develops within the first 5 years of life, up to 90% of cases. In accordance with previous studies, in our clinic center, autistic children are prone to suffering from AD, which impairs their daily life and lowers the quality of life further.

The co-occurrence of autism and atopic dermatitis indicates potentially common mechanisms including shared genetic background, common immunologic dysfunction, and autoimmune process. For those with ASD, their siblings were prone to develop atopic diseases (Dai et al., 2019), and children, who suffered from early AD were more likely to develop subsequent ASD (Billeci et al., 2015; Lee et al., 2016). Meanwhile, in prior studies, ASD children also showed an imbalance in inflammatory cytokine and T-cell subsets (Xu et al., 2018). Besides, in recent years, with both incidence increasing, it has come to the realization that environmental factors play an important role in the development of diseases. Digestive symptoms were more easily observed in autistic children including constipation, diarrhea, bloating, and vomiting (Holingue et al., 2018; Vargason et al., 2019; Lasheras et al., 2020). Compared to neurotypical children, autistic children showed lower bacterial diversity, which was inversely associated with the severity of digestive symptoms (Srikantha and Mohajeri, 2019). There are lots of studies revealing intestinal dysbiosis in ASD and, mostly, showing a decreased ratio of Bacteroidetes to Firmicutes (Srikantha and Mohajeri, 2019). Based on prior studies, Xu et al. conducted a meta-analysis and found that autistic children had lower abundance of *Enterococcus*, *Escherichia coli*, *Bacteroides*, and *Bifidobacterium* and higher abundance of *Lactobacillus* compared to controls (Xu et al., 2019). *Clostridia* species were also found richer in ASD and related to the severity of the autistic condition (Iovene et al., 2017). The emergence of the microbiota–gut–brain axis led to more research on the underlying mechanism and the possible strategies to ameliorate the clinical symptoms. Autistic children presented altered metabolic profiles in urinary and blood analyses, which could be the biomarker of gut dysbiosis (Srikantha and Mohajeri, 2019). Meanwhile, there are several studies revealing promising results that the application of antibiotics and probiotics could improve not only the mental conditions of autistic children but also digestive symptoms (Critchfield et al., 2011; Patusco and Ziegler, 2018; Shaaban et al., 2018; Niu et al., 2019), even though a consensus has not been reached. Similarly, the gut–skin axis described the mutual connection between the gut and the skin, fitting the hygiene hypothesis (Sinha et al., 2021). For those with AD, their gut microbial diversity was lower than that in healthy individuals (Fang et al., 2021). Furthermore, many studies have shown intestinal dysbiosis in children with AD, which could be further improved by probiotics (Watanabe et al., 2003; Roessler et al., 2012; Hulshof et al., 2017). The alteration of the gut microbiota may influence the ratio among T-cell subsets and the metabolic pathway, which induces the inflammatory response and the development of AD.

As aforementioned, the gut microbiota plays an important role in disease development, which could be affected by diet, living habits, and mental stress. Kong et al. enrolled 20 individuals with ASD, and they found an increased relative abundance of gut *Proteobacteria* in ASD with allergy compared with those without allergy, which was not observed in neurotypical controls (Kong et al., 2019). Considering the high prevalence of AD in autism, in this study, we aim to explore the different compositions of gut microbiota between autistic children with and without AD. Besides, we further conduct a non-invasive urinary analysis to detect the possible metabolic changes, which may give some clues to the underlying mechanism of gut microbes on AD development in autism and aid in the clinical management of those special individuals.

## Materials and Methods

### Children

In this cross-sectional study, we enrolled autistic outpatients in Peking University Medical College Hospitals between September 2015 and January 2018. The inclusion criteria were as follows: ① age between 2 and 12 years; ② be diagnosed with ASD in Peking University Sixth Hospital based on the Diagnostic and Statistical Manual of Mental Disorder (Fourth Edition); and ③ the score of Autism Behavior Checklist (ABC) or Childhood Autism Rating Scale (CARS) more than 30 to avoid the potential influence of Attention Deficit Hyperactivity Disorder and other neuropsychiatric diseases. In our study, atopic dermatitis was diagnosed by a professional dermatologist in our hospital according to the criteria of the UK proposed by Williams HC in 1994 (Williams et al., 1994). Moreover, those skin conditions and autoimmune diseases, which mimic atopic dermatitis, were excluded, such as allergic contact dermatitis, seborrheic dermatitis, and psoriasis. Furthermore, those who had no allergy were considered as controls. The Peking Union Medical College Hospital Ethics Committee approved this study, and all participants or their caregivers signed consent forms when enrolled in this clinical study.

### Disease Assessment

A series of questionnaire surveys were conducted to fully assess the severity of autism, including ABC, CARS, Clinical Language State Questionnaire (CLSQ), and The Autism Treatment Evaluation Checklist (ATEC). CLSQ involves two aspects—expression (CLSQ-I) and cognition (CLSQ-II)—and a lower score indicates more severe language impairment. ATEC consists of four subscales, and a higher score is in consistency with the increasing severity of autistic symptoms.

In case of bias, we omitted the item “insensitive to pain” in ATEC, considering that it was hard to be described precisely by children themselves or their caregivers. Meanwhile, these following items potentially relevant to gastrointestinal (GI) problems were added based on clinical experience and scored from 0 to 3 (0 means absence): “feces containing undigested food or smelling sour”, “hiccup/acid regurgitation/abdominal distension”, “extremely happy of unknown reason”, “jump/run back and forth”, and “difficulty in concentrating”.

### Fecal and Urine Collection

Before fecal collection, participants were required not to take any dosage of antibiotics in a month and any probiotics in 2 weeks. Also, fruits and tomatoes were prohibited in 24 h before urine collection. A fresh fecal sample and the first clean midstream specimen of urine in the morning were collected. All samples were stored in dry ice within 3 min since collected.

### Metagenomics Sequencing and Microbial Analysis

We extracted DNA from fecal samples according to the protocol of the MO-BIO PowerSoil DNA Isolation Mini Kit (Carlsbad, CA, USA). Gel electrophoresis was applied for sample quality inspection and quality level judgement. Moreover, the sequencing library construction and template preparation were based on the NEBNext Ultra DNA Library Prep Kit (New England Biolabs, Ipswich, MA, USA). Each sample was marked by a barcode, and equal quantities of barcoded libraries were used for sequencing. All extracted DNA samples were stored at -80°C, and the final sequencing libraries also received quality and quantity assessment before Illumina sequencing. NGS analysis was performed on an Illumina instrument according to the manufacturer’s instructions (Illumina, San Diego, CA, USA). Illumina HiSeq 2500 and HiSeq X Ten sequencing systems (Illumina, CA, USA) were applied for paired-end 150-bp sequencing. In order to appreciate the diversity and to sample a sufficient number of microbial genes, generating at least 4 Gb of data per sample was recommended in our study.

MetaPhlAn (version 1.7.7) (Segata et al., 2012) was used for analyzing the main bacterial taxonomic levels and the relative abundance of the species level. Then, the linear discriminant analysis (LDA) effect size (LEfSe) (Segata et al., 2011)was applied to identify the difference of taxonomic biomarkers between autistic children with and without AD. The Vegan R-package was used for diversity analysis. SOAPaligner (version 2.21) was used to do the alignment and retain the unique mapped reads to do the downstream analysis. We defined the genome-size-normalized relative abundance of these (super)contigs or genomes which was calculated based on the number of aligned reads normalized by the (super)contig’s or genome’s size. We used an integrated non-redundant gene catalog database about the human gut microbiome to do the function analysis (Li et al., 2014) with the Kyoto Encyclopedia of Genes and Genomes (KEGG) database therein.

### Urinary Organic Acid Assay

The urine samples were analyzed by gas chromatography–mass spectrometry, following the instructions described in the study (Shaw et al., 1995). All analyses were conducted at the Great Plains Laboratory, Inc. (Lenexa, KS, USA). A total of 75 metabolites were measured finally, and their concentrations were normalized by the concentration of creatine in the same specimen.

### Statistical Analysis

Continuous variables were expressed as mean and standard deviation or median and quartile if appropriate and further analyzed by Student’s *t*-test or Mann–Whitney U test. Categorical variables were analyzed by *chi*-square test or Fisher’s exact test. For urinary organic acids, those with a P value less than 0.05 in univariate analysis were further enrolled in multivariate binary logistic regression analysis using the method of forward stepwise. Then, the Spearman correlation test was applied to explore the correlation among gut microbiota, their functional pathways, and urinary organic acids. Statistical analyses were performed in SPSS 24.0.0.0 and R software (version 4.0.0), and a two-tailed P value less than 0.05 was considered statistically significant.

## Results

### The Baseline Characteristics of Enrolled Children

A total of 61 children were enrolled in our study between 2015 and 2018, including 36 children in the AD group and 25 children in the control group. The basic characteristics of both groups are shown in **Table 1**. The sex ratio between AD individuals and controls was not statistically significant. The average age of each group was 3.86 ± 2.22 and 4.12 ± 1.83 years, respectively (P = 0.293). The scores of ABC, CARS, CLSQ, and ATEC between both groups did not show statistical significance, which meant the conditions of autism were comparable (**Table S1**).

**Table 1:** The basic characteristics of enrolled autistic children with atopic dermatitis and controls.

	Atopic dermatitisN = 36	Control groupN = 25	P value*
**F/M, n**	6/30	5/20	0.747
**Age, year**	3.86 ± 2.22	4.12 ± 1.83	0.293
**Newly added items related to gastrointestinal symptoms**			
**Feces containing undigested food or smelling sour**	1.44 ± 1.05	0.72 ± 0.94	0.007
**Hiccup/acid regurgitation/abdominal distension**	1.00 ± 0.89	0.60 ± 0.76	0.071
**Extremely happy of unknown reason**	1.50 ± 0.84	1.12 ± 0.93	0.095
**Jump/run back and forth**	1.64 ± 0.80	1.20 ± 0.87	0.065
**Difficulty in concentrating**	1.94 ± 0.75	2.04 ± 0.89	0.499

F, female; M, male.

Continuous variables were expressed as mean and standard deviation.

*Statistical test: Mann–Whitney U test for numerical data and Fisher’s exact test for categorial data.

Considering the verbal limitation in children, we newly added five items in our study, which could be the indicators of potential GI problems to some extent. According to **Table 1**, these children in the AD group had a more severe condition of feces containing undigested food or smelling sour (1.432 ± 1.042 vs. 0.720 ± 0.936, P = 0.007). Meanwhile, we also compared the fourth subscale of ATEC about behavior (data not shown) and found that AD children were more likely to be unhappy or crying compared with those without AD (1.162 ± 0.688 vs. 0.640 ± 0.638, P = 0.004).

### The Difference in Compositions and Functional Profiles of Gut Microbiota

**Figure 1:**
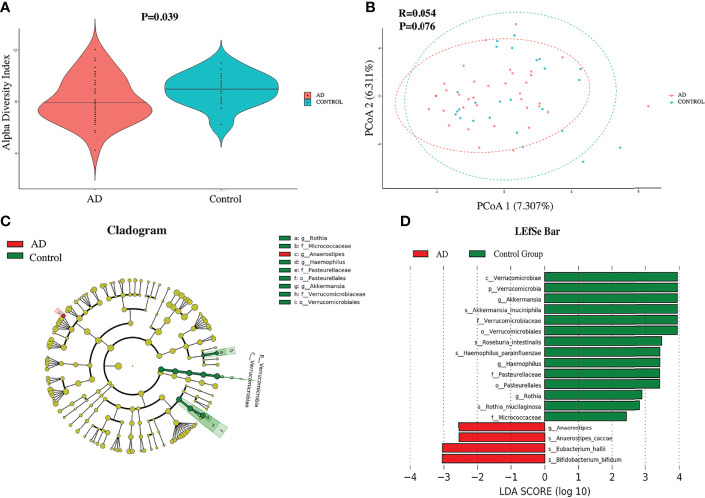
The diversity and compositions of gut microbiota between autistic children with and without atopic dermatitis. **(A)** The violin plot of the alpha diversity index between two groups. **(B)** Principal coordinate analysis. **(C)** Red and green dots showed the relatively abundant bacterial taxa in AD individuals and the control group, respectively. Concentric rings from inside to outside were phylum, class, order, family, and genus. **(D)** Linear discriminant analysis (LDA) effect size (LEfSe) of gut microbiota. Those richer in AD individuals are represented in the red bar with a negative LDA score, and the control group in the green bar with a positive score. Only items with an absolute LDA value more than 2 are shown.

**Figure 2:**
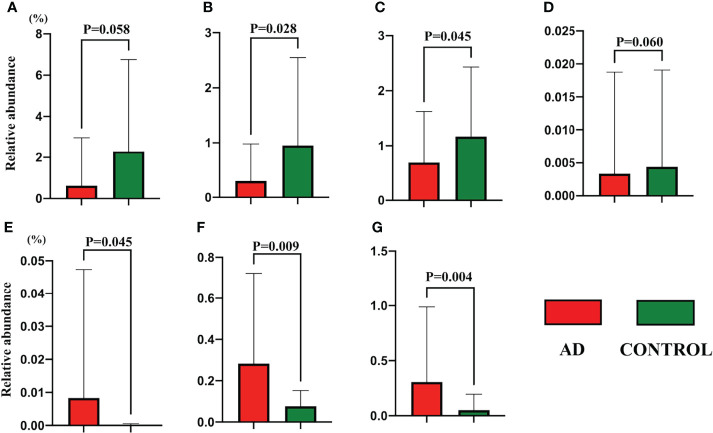
The relative abundance of gut species detected in the LEfSe analysis between autistic children with and without atopic dermatitis. **(A)** *Akkermansia muciniphila*; **(B)** *Roseburia intestinalis*; **(C)** *Haemophilus parainfluenzae*; **(D)** *Rothia mucilaginosa*; **(E)** *Anaerostipes caccae*; **(F)** *Eubacterium hallii*; **(G)** *Bifidobacterium bifidum*. Data are shown in mean and standard deviation, and the Mann–Whitney U test was applied.

The relevant analyses of gut microbiota are presented in **Figure 1**. For alpha diversity, AD individuals showed a lower alpha diversity index than their control counterparts (P = 0.039) (**Figure 1A** **)**, while there was no significant difference in terms of Pielou’s evenness, Shannon Diversity Index, and Simpson’s Diversity Index (P = 0.9, 0.87, and 0.82, respectively). Additionally, principal coordinate analysis (PCoA) could not make a distinction between individuals in the AD group and those in the control group (**Figure 1B**). The LEfSe method was applied to identify the phylotypes abundant in these two groups (**Figures 1C, D**). A significant increase of *Anaerostipes caccae* and *Eubacterium hallii* was found in AD individuals (**Figure 1D**), and *Roseburia intestinalis* was abundant in controls. Intriguingly, *Bifidobacterium bifidum* was richer in AD individuals. Moreover, *Akkermansia muciniphila*, *Haemophilus parainfluenzae*, and *Rothia mucilaginosa* were positively associated with controls. The relative abundance of above species is presented in **Figure 2**.

**Figure 3:**
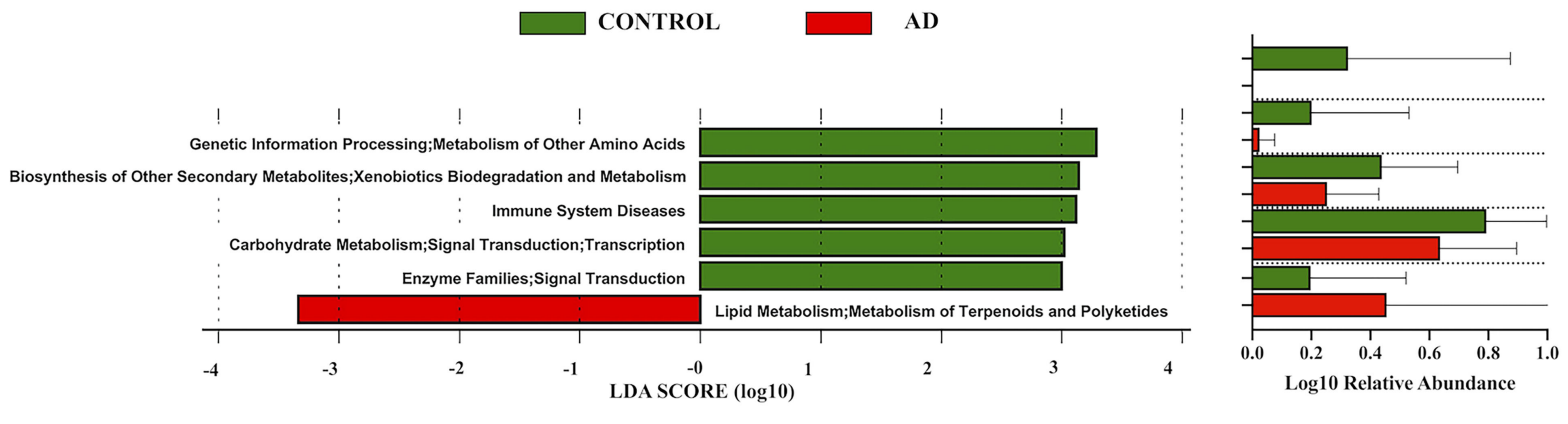
Linear discriminant analysis (LDA) effect size (LEfSe) of functional profiles between autistic children with and without atopic dermatitis. Only items with an absolute LDA value more than 2 are shown.

Then pathway analyses (**Figure 3**) revealed that the pathways of carbohydrate metabolism, metabolism of amino acids, and xenobiotics biodegradation were prominent in control individuals, while the pathway of lipid metabolism showed a predilection in the AD group.

### The Increase of Urinary Organic Acids in AD Individuals

A total of 75 organic acids were reported from the spectrum analysis. PCoA did not show an obvious demarcation between AD individuals and controls (data not shown). Herein, we only presented those items with statistical significance based on the univariate test (**Table 2**). Others are presented in **Table S2**. Among urinary organic acids, adipic acid, 3-hydroxyglutaric acid, tartaric acid, homovanillic acid, 2-hydroxyphenylacetic acid, aconitic acid, and 2-hydroxyhippuric acid had higher concentrations in the AD group. However, only adipic acid was enrolled in the final logistic regression model (OR = 1.513, 95% CI [1.042, 2.198], P = 0.030).

**Table 2:** The comparative results of urinary organic acids between children with atopic dermatitis and without (controls).

Items	Controls N = 25	Atopic dermatitis N = 36	P value
**Adipic acid**	2.005 ± 1.669	3.240 ± 2.147	0.002
**3-Hydroxyglutaric acid**	5.352 ± 2.238	6.703 ± 2.620	0.011
**Tartaric acid**	0.534 ± 0.777	2.033 ± 7.087	0.014
**Homovanillic acid**	4.136 ± 1.686	5.697 ± 3.058	0.016
**2-Hydroxyphenylacetic acid**	0.385 ± 0.201	0.488 ± 0.196	0.030
**Aconitic acid**	11.672 ± 5.202	14.158 ± 4.825	0.046
**2-Hydroxyhippuric acid**	0.616 ± 0.414	1.438 ± 2.057	0.048

Unit: mmol/mmol creatinine. Just those with a two-sided P value less than 0.05 in the Mann–Whitney U test are shown. Data were presented as mean and standard deviation.

### The Correlation Analysis

**Figure 4:**
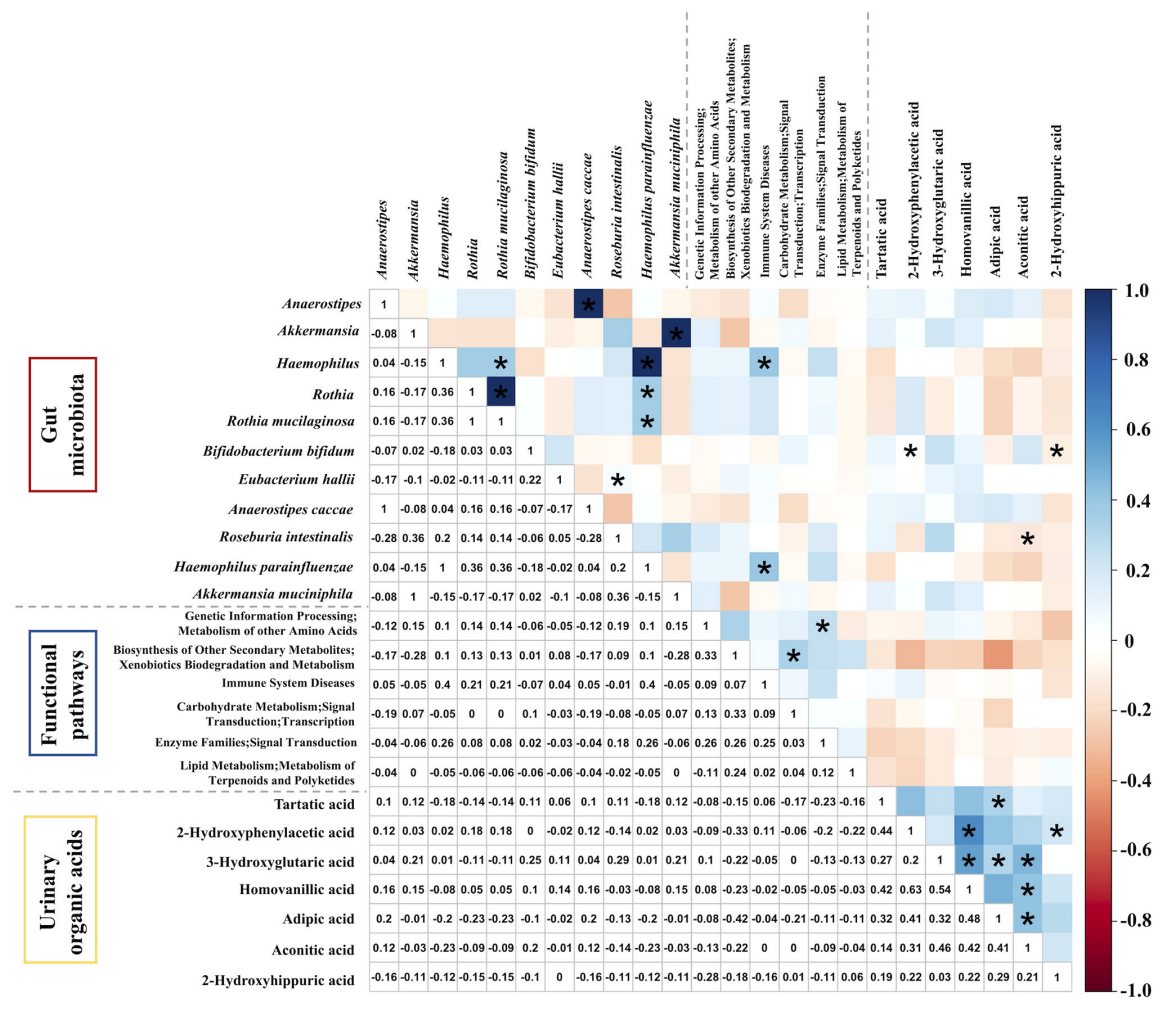
The Spearman correlation matrix among gut microbiota, functional pathways, and urinary organic acids different in autistic children with and without atopic dermatitis. *P < 0.05.

The Spearman correlation test was applied to further explore the correlation among gut microbiota; functional pathways (LEfSe: P < 0.05, LDA >2) with those urinary organic acids were significant in univariate analysis (**Figure 4**). According to the matrix, *Roseburia intestinalis*, enriched in those without atopic dermatitis, had a negative correlation with aconitic acid (r = -0.14, P = 0.02). As for the functional pathways and urinary organic acids, however, we did not observe any statistically significant correlation. The pathway of xenobiotics biodegradation and metabolism seems to inversely correlate with adipic acid (r = -0.42), yet with a P value of 0.18. Among urinary organic acids, aconitic acid was positively correlated with adipic acid (r = 0.41, P = 0.006). Besides, there were positive correlations among urinary organic acids with a correlation coefficient of about 0.5. This could directly explain why the difference in results existed between logistic regression and univariate analysis for urinary organic acids. We also conducted correlation analysis between other taxa at genus or species level identified in metagenomic sequencing and those aforementioned urinary organic acid, as shown in **Figures S1, 2**. Nevertheless, no strong correlation was further revealed.

## Discussion

The coexistence of atopic dermatitis in autism is far from rare, which bothers those children and further lowers their life quality. This is the first study exploring the alterations of gut microbiota and urinary organic acids in autistic children with AD compared with those without AD. We found that there was no significant difference in sex, age, and disease evaluation between both groups, which was reasonable to carry out further analysis.

As expected, in our study, autistic children with AD had more prominent GI symptoms than their control counterparts. GI symptoms showed greater prevalence in patients with ASD compared to those without ASD (McElhanon et al., 2014). As for patients with AD, lots of studies have revealed that AD is related to the increased risk of GI disorders including Crohn’s disease, ulcerative colitis, and celiac disease (Baron et al., 2005; Ress et al., 2014; Augustin et al., 2015). Considering the limitation of verbal communication in children, their underlying GI problems may be presented as sleep or mood disturbance (Maenner et al., 2012). In our study, AD individuals had a higher score in the item about being unhappy or crying than that in controls.

In the analysis of gut microbiota, lower diversity was observed in the AD group, which was consistent with previous studies on autism and atopic dermatitis (Petersen et al., 2019; Bezawada et al., 2020). This may echo the known theory—the hygiene hypothesis—in which the lack of exposure to a high diversity of microbes in early life is related to a higher prevalence of chronic inflammatory disorders as well as psychiatric disorders (Garn et al., 2021). In our study, *B. bifidum* showed an enrichment in autistic children with AD (0.31% vs. 0.05%). For children with ASD, Xu et al. summarized that the relative abundance of *Bifidobacterium* was lower compared to controls (Xu et al., 2019). Even though several studies had revealed that *Bifidobacterium* was less in AD infants compared to healthy controls (Fang et al., 2021), controversy always exists. A cross-sectional study from Brazil revealed that AD children had a greater abundance of *Bifidobacterium* (OR: 11.09; 95% CI: 2.14; 57.39) (Melli et al., 2020). At species levels, Waligora-Dupriet et al. enrolled 10 allergic infants and 20 controls, and they did not detect any alteration in the diversity of gut *Bifidobacterium* species (Waligora-Dupriet et al., 2011). However, Suzuki et al. found that *B. bifidum* had a higher prevalence in allergic infants than healthy controls (70% vs. 12.5%, P < 0.01), which was irrelevant to feeding methods (Suzuki et al., 2007). Furthermore, Huang and coauthors enrolled 13 randomized controlled trials and found it was not robust to conclude that probiotics were beneficial to children with AD (Huang et al., 2017). Similarly, Yang et al. did not observe the therapeutic or immunomodulatory effects of probiotics on the treatment of AD (Yang et al., 2014). Additionally, as mentioned above, as a possible comorbidity of AD, Nistal et al. observed that the diversity of *Bifidobacterium* species was reduced in treated patients with celiac disease while *B. bifidum* was enriched in untreated patients (Nistal et al., 2012). Therefore, it is necessary to further investigate the relationship between conventional probiotics and the development of AD in autistic children.

In this present study, we observed different distributions of species from family *Lachnospiraceae* in both groups, among which *A. caccae* and *E. hallii* predominated in AD individuals, with *R. intestinalis* in controls (8.22 × 10^-4^ vs. 1.18 × 10^-5^; 0.28% vs. 0.08%; 0.30% vs. 0.95%). All these microbes were involved in the production of short-chain fatty acids (SCFAs), such as acetate, propionate, and butyrate (Louis et al., 2014). SCFAs, the major products from the fermentation activity of gut microbes, are proved to play a crucial role in host health (Wong et al., 2006; Koh et al., 2016; Sivaprakasam et al., 2016; Gill et al., 2018; Hu et al., 2018). They could promote the differentiation of regulatory T cells and the production of the anti-inflammatory cytokines, avoiding colonic inflammation and cancer development, as well as modulate host energy metabolism (Sleeth et al., 2010; Singh et al., 2014; Koh et al., 2016; Hu et al., 2018). Additionally, it became increasingly accepted that SCFAs could mediate epigenetic modification, which was negatively associated with allergic sensitization and events (Acevedo et al., 2021). Reddel et al. showed that AD children were characterized as a reduction of SCFA-producing bacteria (Reddel et al., 2019). Moreover, the severity of atopic eczema was inversely correlated with the abundance of butyrate-producing bacteria (r = -0.52, P = 0.005) (Nylund et al., 2015). Meanwhile, in an ovalbumin-induced mouse model of AD, the use of antibiotics before primary ovalbumin sensitization would reduce gut SCFA levels and aggravate AD symptoms mediated by gut dysbiosis (Kim et al., 2020). Nevertheless, in autism, SCFA is a double-edged sword. Compared to healthy controls, autistic children had a lower concentration of SCFAs, among which the level of butyrate was dramatically reduced while propionate and acetate were in higher levels (De Angelis et al., 2013). The injection of propionate into rats could cause ASD-like symptoms, and it was associated with *Clostridia* species, which was generally acknowledged involved in the pathophysiology of ASD (Srikantha and Mohajeri, 2019). Butyrate is considered as the most important SCFA in hosts. Lots of studies had shown its role in brain protection and its positive effects on neurodegenerative diseases (Mohajeri et al., 2018).

*R. intestinalis*, a butyrate-producing bacterium in the colon, has come to the fore due to its role in the prevention of intestinal inflammation and the maintenance of host homeostasis in many digestive, autoimmune, and neurological diseases (Nie et al., 2021). Of note, one of the fermentative products of *B. bifidum* is lactate (de Vries et al., 1967), which could be the substrate for producing butyrate by *A. caccae* and *E. hallii* besides acetate, while *R. intestinalis* just uses acetate for producing butyrate (Koh et al., 2016). Additionally, *B. bifidum* is the only species to degrade mucin into monosaccharides in genus *Bifidobacterium* and enable the growth of *E. hallii* to produce SCFAs by cross-feeding (Bunesova et al., 2017). To some extent, these may explain why two SCFA-producing bacteria were richer in AD individuals, unexpectedly in our study.

As a promising probiotic, *A. muciniphila* was found richer in autistic children without AD compared to those with AD (2.31% vs. 0.61%). According to previous studies, compared to healthy children, atopic children showed a significant decrease in or even deletion of *A. muciniphila (* Candela et al., 2012; Drell et al., 2015; Sung et al., 2022). Lee et al. found that the colonization of *A. muciniphila* reduced in infants with AD compared to the control group (Lee et al., 2018). Besides atopy, the lower relative abundance of *A. muciniphila* in the gut was also reported to be related to autism (Wang et al., 2011), even though the relative abundance of genus *Akkermansia* was not significantly different between ASD infants and healthy controls (Inoue et al., 2016). Characterized by mucolytic ability, *A. muciniphila* could degrade host mucin to promote the production of SCFAs and regulate the immune system, maintaining the integrity of the gut barrier (Belzer et al., 2017; Ottman et al., 2017; Zhang et al., 2019; Zhou and Zhang, 2019). Meanwhile, SCFAs should bind to G protein-coupled receptors to achieve the normal resolution of inflammatory response (Maslowski et al., 2009), the expression of which was regulated by *A. muciniphila (* Lukovac et al., 2014). Compared to children with persistent AD, the proportion of *Akkermansia* was higher in transient AD cases, indicating its potential role in the remission of AD (Park et al., 2020).

Surprisingly, we found that the amounts of *H. parainfluenzae* and *R. mucilaginosa* were significantly higher in controls compared with AD individuals (1.17% vs. 0.68%; 4.42 × 10^-5^ vs. 3.28 × 10^-5^). Both two species are usually regarded as opportunistic pathogens, which could lead to a variety of diseases, such as pneumonia, endocarditis, and bacteremia (Spernoga, 1980; Bruminhent et al., 2013; Maraki and Papadakis, 2015; Cobo et al., 2017; Faure et al., 2017; Poyer et al., 2019). However, Zheng and coauthors identified that genus *Haemophilus* was richer in healthy infants compared to infants with eczema (Zheng et al., 2016). In a study by Arrieta et al., the reduced amount of genus *Rothia* in the gut during the first 100 days of life was associated with a higher risk of childhood asthma by means of reducing the level of fecal acetate (Arrieta et al., 2015). In ASD, Kang et al. revealed that a decrease in the abundance of *H. parainfluenzae* in the gut was related to more severe GI symptoms in children with ASD (Kang et al., 2018), similar to the study conducted by Zou et al. (2020). By far, no study has yet been reported on the relationship between these two species and AD.

The gut dysbiosis may come along with the disruption of the host barrier and the alteration of metabolism, which could be further reflected by the metabolic analysis in feces, blood, and urine. In our study, for urinary organic acids, only adipic acid was statistically significant in the multivariate analysis (OR = 1.513, P = 0.030). Adipic acid was produced from the omega-oxidation pathway of fatty acids, a process which was normally an alternative to the beta-oxidation pathway in mitochondria (Puig-Alcaraz et al., 2016). This pathway could be augmented when mitochondrial function was impaired. Compared to healthy controls, the increase of adipic acid in urine was significantly correlated with ASD and its severity (Puig-Alcaraz et al., 2016; Khan et al., 2022). Furthermore, in the correlation analysis, aconitic acid and adipic acid were positively related. Aconitic acid was an intermediate product in the Krebs cycle and produced by the dehydration citric acid (Mussap et al., 2016). The increase of aconitic acid in urine was observed in individuals with autism, depression, or autoimmune diseases compared to healthy controls (Jones et al., 2005; Noto et al., 2014). Both indicated mitochondrial dysfunction. Mitochondria critically modulated host immune response, and its dysfunction could induce oxidative stress favoring systemic inflammation, which was observed to be involved in allergic diseases including atopic dermatitis, allergic rhinitis, and asthma (Iyer et al., 2017; Trinchese et al., 2018). Zhang et al. found that the deficiency of the detoxification pathway of the gut microbiota was linked to the biomarkers of mitochondrial dysfunction (Zhang et al., 2020). This was in line with the enrichment of the pathway of xenobiotics biodegradations in the control group, and this pathway of the gut microbiota seems to have a negative correlation with adipic acid (r = -0.42, P = 0.18). Besides, we also found that *R. intestinalis* showed a negative correlation with aconitic acid, which was the same with their distributions in different groups. The exposure of toxic substances may contribute to mitochondrial dysfunction (Lee et al., 2010), finally leading to disease development. Meanwhile, with the dramatical increase in the incidence of AD, epigenetic regulation was brought to the fore and considered as a link between the changing environment and genetic changes, which had been explored extensively (Sroka-Tomaszewska and Trzeciak, 2021). The disorders of xenobiotic biodegradation may increase the risk of environmental exposure and detrimental epigenetic modifications, shifting the host toward a higher risk of allergic diseases. The specific mechanisms of gut dysbiosis on AD development in autism require more evidence in the future.

Besides, as **Table 2** shows, there were some urinary organic acids, known for the markers of metabolic disorders in amino acids, enriching in the AD group, including 3-hydroxyglutaric acid (Al-Dirbashi et al., 2011) and 2-hydroxyphenylacetic acid (Armstrong et al., 1955), even though both have become statistically insignificant in the multivariate analysis. This was consistent with the result that the pathway of amino acid metabolism was found predominant in the control group. Autistic children showed a perturbation of phenylalanine metabolism, with elevated concentrations of 3-(3-hydroxyphenyl)-3-hydroxypropionic acid, 3-hydroxyphenylacetic acid, and 3-hydroxyhippuric acid in urinary analysis (Xiong et al., 2016). Similarly, 2-hydroxyhippuric acid was richer in the AD group, which was produced in the liver during the process of detoxification of salicylic acid (Suh et al., 1986). Its level generally increased with the intake of exogenous salicylic acid such as aspirin. Nevertheless, Finnie et al. enrolled children with digestive symptoms, to whom no salicylate-containing drugs were administrated. They found that 2-hydroxyhippuric acid was detected in the urine of sick children and its level was associated with GI disorders (Finnie et al., 1976). Considering that salicylic acid showed an anti-inflammatory effect in colitis (Miquel et al., 2015), it was reasonable to deduce that the production of salicylic acid may rise with the severity of GI disorders owning to self-defense mechanisms, and we really observed more GI dysfunctions in autistic children with AD.

We acknowledge that our study has several limitations. Firstly, it was a single-center study and the sample size was limited. Secondly, in this study, we only enrolled autistic children with AD and without AD, lacking those healthy or only with AD as controls. It is impossible for us to exclude the potential effect of autism as a confounder. Thirdly, due to the limitation of technology, we just took urinary organic acids into consideration, not including fecal or blood samples, which may affect the final result to some extent. Apart from the complex network among gut microbiota and their interactions with the host, this may explain why we did not observe evident correlations between urinary organic acids and gut microbiota. Lastly but very importantly, we just limited the usage of antibiotics and probiotics before sample collection, while the diets of enrolled children were not obtained and analyzed, which would introduce deviation in the final results. The question of causality of the alteration of the gut microbiota and the development of AD remains an egg or chicken problem. All of these will make our study preliminary and exploratory. Large-scale and well-designed studies in the future are needed.

Altogether, lots of studies have shed light on the role of the gut microbiota in the pathogenesis of disease. Herein, we identified significant differences of gut microbiota and urinary organic acids between autistic children with and without AD, and the underlying mechanism on AD occurrence in autism needs further investigation. A more comprehensive understanding of the relationship between gut microbiota and AD in autistic children will be beneficial for clinicians to manage those children in the prevention and treatment of AD better.

## Data Availability Statement

The data presented in the study are deposited in the National Center for Biotechnology Information (NCBI) Bioproject repository, accession number PRJNA845014.

## Ethics Statement

The studies involving human participants were reviewed and approved by the Peking Union Medical College Hospital Ethics Committee. Written informed consent to participate in this study was provided by the participants’ legal guardian/next of kin.

## Author Contributions

All authors contributed to the study conception and design. Material preparation and data collection were performed by XY, X-jX, X-fZ, and XZ. Data analysis and interpretation were performed by R-pH, Y-yH, GT, Y-fJ, and J-dL. The first draft of the manuscript was written by R-pH, Y-yH, and X-jX, and all authors commented on previous versions of the manuscript. All authors contributed to the article and approved the submitted version.

## Funding

This work was supported by the Autism Special Fund from the Peking Union Medical Foundation, the CAMS Innovation Fund for Medical Science (CIFMS) (2017-I2M-3-017), and the Non-profit Central Research Institute Fund of Chinese Academy of Medical Sciences (2019XK320030). The study sponsors had no involvement in the study design, the collection, analysis, and interpretation of data, the writing of the report, and the decision to submit the paper for publication.

## Conflict of Interest

Authors J-dL and GT were employed by the company Geneis (Beijing) Co., Ltd.

The remaining authors declare that the research was conducted in the absence of any commercial or financial relationships that could be construed as a potential conflict of interest.

## Publisher’s Note

All claims expressed in this article are solely those of the authors and do not necessarily represent those of their affiliated organizations, or those of the publisher, the editors and the reviewers. Any product that may be evaluated in this article, or claim that may be made by its manufacturer, is not guaranteed or endorsed by the publisher.
