# Supplementary figures and images for: The Difference of Gut Microbiota and Their Correlations With Urinary Organic Acids Between Autistic Children With and Without Atopic Dermatitis
Source: Front Cell Infect Microbiol. 2022 Jun 21;12:886196. doi: 10.3389/fcimb.2022.886196 (PMC9253573; doi:10.3389/fcimb.2022.886196)

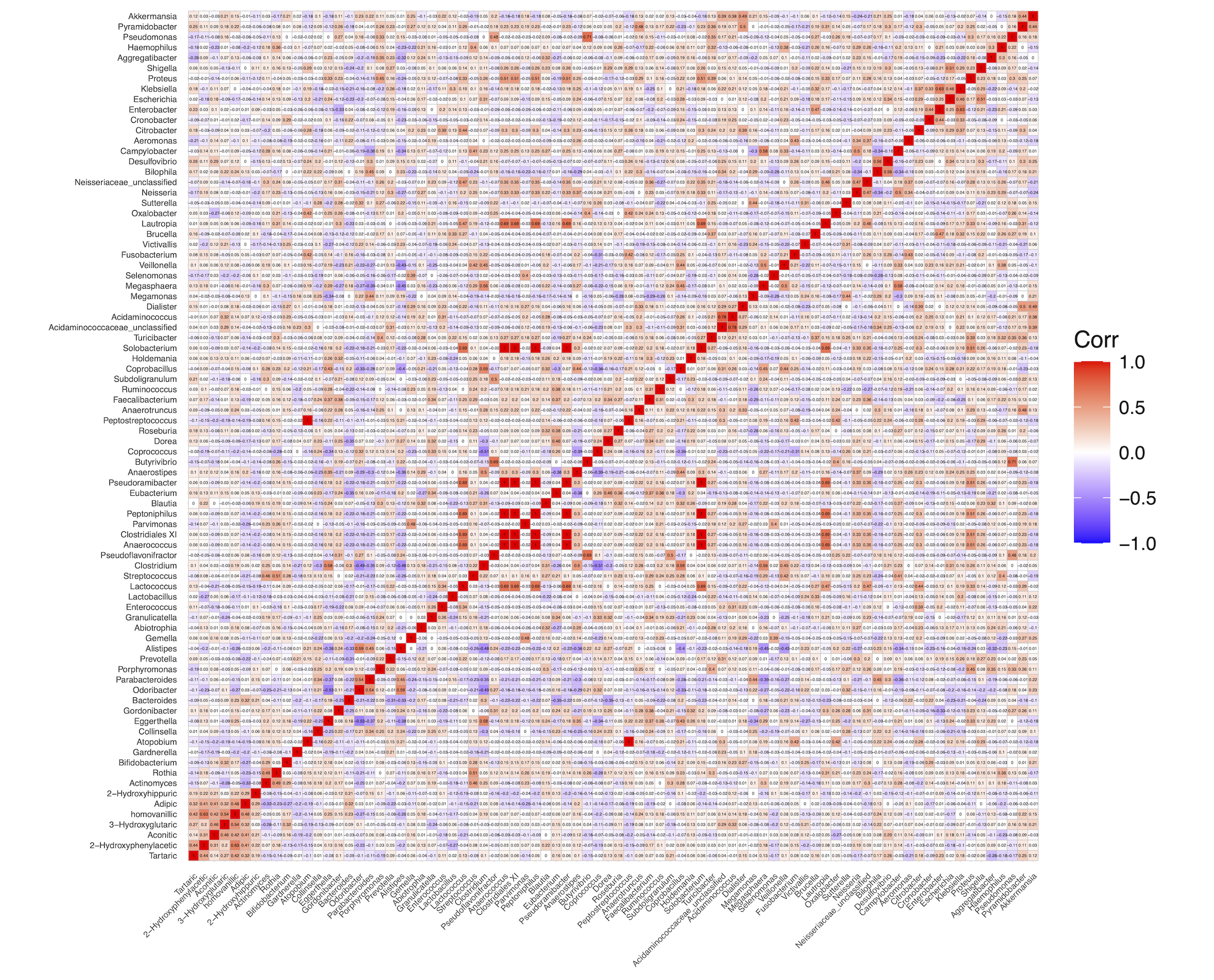

Supplement: Supplementary file 1 [file Image_1.tiff]

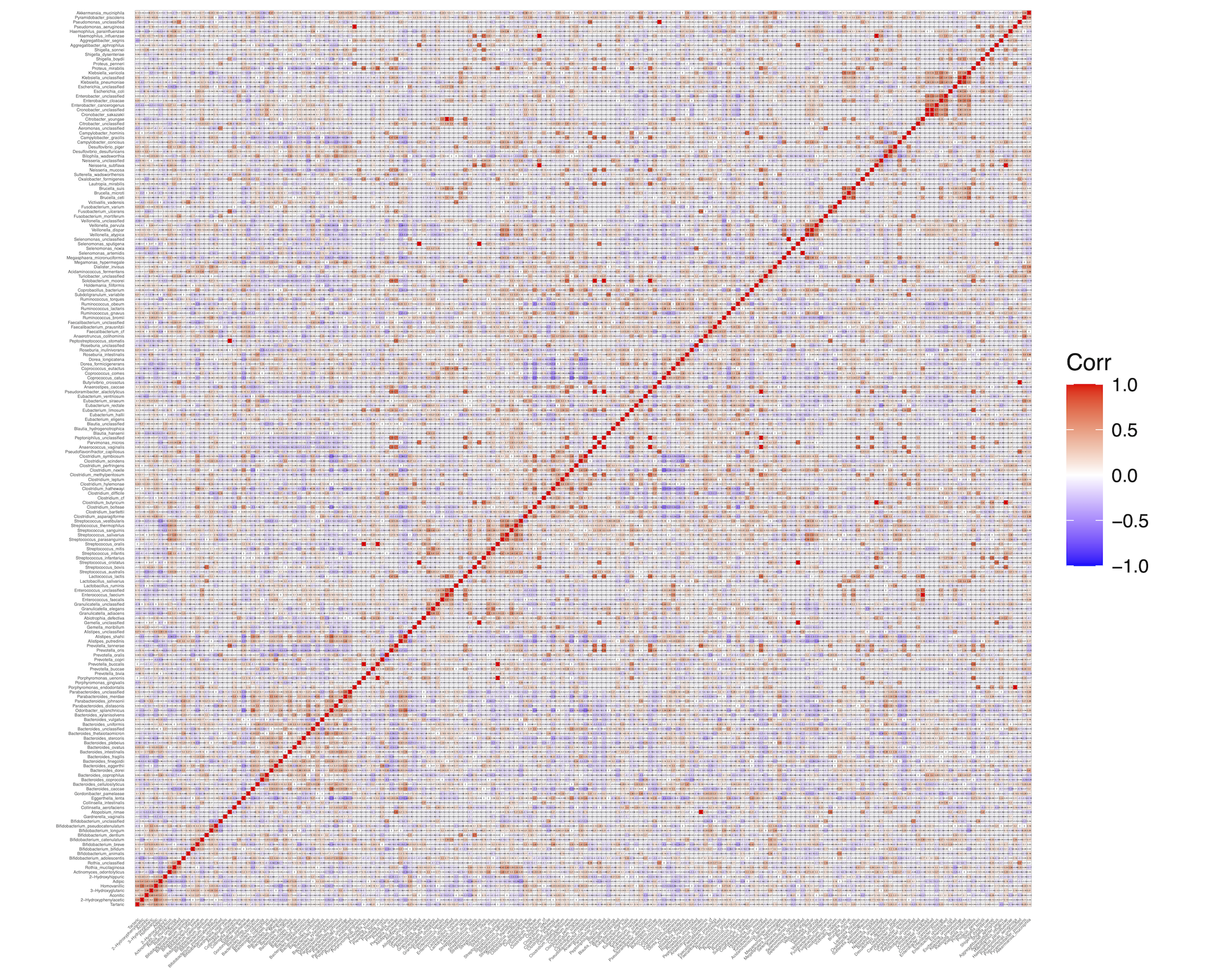

Supplement: Supplementary file 2 [file Image_2.tiff]
