# Supplementary material for: The Difference of Gut Microbiota and Their Correlations With Urinary Organic Acids Between Autistic Children With and Without Atopic Dermatitis
Source: Front Cell Infect Microbiol. 2022 Jun 21;12:886196. doi: 10.3389/fcimb.2022.886196 (PMC9253573; doi:10.3389/fcimb.2022.886196)
Supplement: Supplementary file 3 [file Table_1.docx]

|  | **Atopic dermatitis**  **N=36** | **Control group**  **N=25** | **P value** |
| --- | --- | --- | --- |
| **ABC** | 57.00±20.81 | 56.16±21.51 | 0.849 |
| **CARS** | 33.83±6.78 | 33.40±6.20 | 1.000 |
| **CLSQ** | 9.61±5.15 | 11.88±5.68 | 0.112 |
| **CLSQ-I** | 4.31±3.16 | 5.92±3.33 | 0.059 |
| **CLSQ-II** | 5.31±2.34 | 5.96±2.65 | 0.145 |
| **ATEC** | 81.06±23.05 | 69.72± 25.83 | 0.099 |

**Table S1. The severity assessment of autism of enrolled autistic children with atopic dermatitis and controls.**

ABC, Autism Behavior Checklist; CARS, Childhood Autism Rating Scale; CLSQ, Clinical Language State Questionnaire; CLSQ-I, “Expression” Part in CLSQ; CLSQ-II, “Cognition” Part in CLSQ; ATEC, Autism Treatment Evaluation Checklist.

Data was expressed as mean and standard deviation.
