# Supplementary material for: The Difference of Gut Microbiota and Their Correlations With Urinary Organic Acids Between Autistic Children With and Without Atopic Dermatitis
Source: Front Cell Infect Microbiol. 2022 Jun 21;12:886196. doi: 10.3389/fcimb.2022.886196 (PMC9253573; doi:10.3389/fcimb.2022.886196)
Supplement: Supplementary file 4 [file Table_2.docx]

**Table S2: T****he comparative results of urinary organic acids between children with atopic dermatitis and without (control group).**ˉ

| **Items** | **Atopic dermatitis**  **N=36** | **Controls**  **N=25** | **P value****†** |
| --- | --- | --- | --- |
| **Adipic** | 3.24±2.147 | 2.005±1.669 | 0.002 |
| **3-Hydroxyglutaric** | 6.703±2.62 | 5.352±2.238 | 0.011 |
| **Tartaric** | 2.033±7.087 | 0.534±0.777 | 0.014 |
| **Homovanillic（HVA）** | 5.697±3.058 | 4.136±1.686 | 0.016 |
| **2-Hydroxyphenylacetic** | 0.488±0.196 | 0.385±0.201 | 0.03 |
| **Quinolinic / 5-HIAA Ratio** | 10.661±9.934 | 7.45±8.392 | 0.039 |
| **Aconitic** | 14.158±4.825 | 11.672±5.202 | 0.046 |
| **2-Hydroxyhippuric** | 1.438±2.057 | 0.616±0.414 | 0.048 |
| **Citramalic** | 1.701±0.903 | 1.294±0.847 | 0.05 |
| **Fumaric** | 0.555±1.055 | 0.281±0.177 | 0.054 |
| **2-Oxoglutaric** | 25.086±13.376 | 18.105±9.21 | 0.055 |
| **Malic** | 0.887±0.552 | 0.614±0.377 | 0.058 |
| **Quinolinic** | 5.075±2.072 | 4.252±2.204 | 0.066 |
| **Carboxycitric** | 7.692±15.9 | 9.437±15.735 | 0.071 |
| **3-Hydroxy-3-methylglutaric** | 29.602±22.547 | 21.101±11.742 | 0.072 |
| **5-Hydroxyindoleacetic**  **（5-HIAA）** | 0.798±0.764 | 1.144±1.068 | 0.078 |
| **3-Methylglutaric** | 0.43±0.264 | 0.356±0.259 | 0.086 |
| **3-Methylglutaconic** | 1.855±0.88 | 1.498±0.684 | 0.094 |
| **N-Acetylaspartic** | 1.622±1.612 | 2.268±1.622 | 0.094 |
| **Orotic** | 0.629±0.515 | 0.443±0.208 | 0.097 |
| **HPHPA** | 95.905±127.479 | 41.336±34.715 | 0.125 |
| **Vanillylmandelic（VMA）** | 2.907±1.187 | 2.436±0.881 | 0.127 |
| **Sebacic** | 0.178±0.289 | 0.113±0.115 | 0.129 |
| **DHPPA** | 0.385±0.614 | 0.188±0.223 | 0.13 |
| **4-Hydroxyhippuric** | 16.163±13.123 | 11.564±7.97 | 0.135 |
| **2-Hydroxybutyric** | 1.315±0.794 | 1.728±1.195 | 0.14 |
| **5-Hydroxymethyl-2-furoic** | 5.156±6.753 | 3.041±3.202 | 0.163 |
| **Furan-2,5-dicarboxylic** | 5.323±7.436 | 2.902±3.175 | 0.163 |
| **Tricarballylic** | 0.247±0.258 | 0.394±0.441 | 0.166 |
| **HVA/VMA** | 2.219±1.576 | 1.784±0.64 | 0.167 |
| **Malonic** | 5.825±4.287 | 4.236±3.081 | 0.172 |
| **Acetoacetic** | 17.317±41.171 | 38.902±126.817 | 0.174 |
| **Citric** | 153.638±107.464 | 117.12±71.036 | 0.175 |
| **4-Cresol** | 24.143±29.24 | 43.768±89.89 | 0.187 |
| **N-Acetylcysteine** | 0.087±0.148 | 0.04±0.067 | 0.187 |
| **Hippuric** | 214.527±169.519 | 146.36±102.693 | 0.189 |
| **Pyroglutamic** | 41.361±19.607 | 34.48±13.635 | 0.21 |
| **3-Oxoglutaric** | 0.107±0.106 | 0.073±0.091 | 0.217 |
| **3-Indoleacetic** | 1.268±0.674 | 1.589±1.097 | 0.223 |
| **3-Methyl-2-oxovaleric** | 0.374±0.334 | 0.499±0.398 | 0.227 |
| **4-Hydroxyphenyllactic** | 0.421±0.224 | 0.372±0.214 | 0.24 |
| **Methylsuccinic** | 2.77±4.448 | 1.861±1.067 | 0.277 |
| **2-Oxoisocaproic** | 0.106±0.078 | 0.144±0.117 | 0.316 |
| **Methylcitric** | 1.121±0.601 | 0.96±0.609 | 0.329 |
| **4-Hydroxybutyric** | 1.534±1.181 | 1.997±1.857 | 0.329 |
| **Uracil** | 7.824±5.361 | 6.22±2.964 | 0.34 |
| **4-Hydroxyphenylacetic** | 21.644±15.983 | 17.032±10.458 | 0.386 |
| **2-Oxo-4-methiolbutyric** | 0.077±0.064 | 0.094±0.078 | 0.386 |
| **4-Hydroxybenzoic** | 1.45±1.128 | 1.12±0.584 | 0.391 |
| **Kynurenic** | 1.645±0.953 | 1.761±0.794 | 0.407 |
| **Pantothenic** | 3.433±2.555 | 2.773±1.756 | 0.411 |
| **Oxalic** | 237.222±169.123 | 273.12±193.666 | 0.455 |
| **3-Hydroxybutyric** | 8.191±15.332 | 9.401±22.232 | 0.5 |
| **Pyridoxic** | 3.005±3.124 | 3.966±4.521 | 0.509 |
| **Glutaric** | 0.678±0.479 | 0.584±0.36 | 0.509 |
| **2-Hydroxyisovaleric** | 0.016±0.063 | 0.092±0.46 | 0.538 |
| **2-Hydroxyisocaproic** | 0.017±0.03 | 0.014±0.038 | 0.574 |
| **Phenyllactic** | 0.057±0.048 | 0.052±0.054 | 0.585 |
| **Methylmalonic** | 1.816±0.907 | 2.244±1.605 | 0.607 |
| **Pyruvic** | 4.54±2.964 | 5.521±4.813 | 0.623 |
| **Furancarbonylglycine** | 0.461±0.564 | 0.323±0.287 | 0.633 |
| **Homogentisic** | 0.028±0.032 | 0.028±0.037 | 0.644 |
| **Mandelic** | 0.116±0.09 | 0.123±0.078 | 0.652 |
| **Thymine** | 0.268±0.16 | 0.248±0.132 | 0.665 |
| **Ethylmalonic** | 2.279±1.391 | 2.111±1.196 | 0.697 |
| **Phenylpyruvic** | 1.385±0.762 | 1.359±0.841 | 0.736 |
| **Lactic** | 21.891±14.766 | 22.612±15.852 | 0.752 |
| **Arabinose** | 70.366±44.108 | 66.428±43.621 | 0.797 |
| **Succinic** | 9.677±7.96 | 7.488±4.42 | 0.82 |
| **Ascorbic** | 26.497±89.63 | 3.152±4.859 | 0.855 |
| **Phosphoric** | 2772.527±1530.104 | 2673.08±993.851 | 0.866 |
| **Suberic** | 4.895±3.855 | 5.423±5.254 | 0.93 |
| **2-Oxoisovaleric** | 0.098±0.211 | 0.113±0.206 | 0.93 |
| **Glycolic** | 155.861±123.775 | 165.32±118.861 | 0.977 |
| **Glyceric** | 3.512±1.47 | 3.892±1.965 | 0.994 |

Data was presented as mean and standard derivation and the unit of the concentration of organic acids was mmol/mol creatinine; †Mann-Whitney U test
